# Formononetin Inhibits Progression of Endometriosis via Regulation of p27, pSTAT3, and Progesterone Receptor: In Vitro and In Vivo Studies

**DOI:** 10.3390/nu15133001

**Published:** 2023-06-30

**Authors:** Yunjeong Park, Sung Pil Choo, Gee Soo Jung, Sehee Kim, Min Jung Lee, Wooseok Im, Hyemin Park, Inha Lee, Jae Hoon Lee, Sihyun Cho, Young Sik Choi

**Affiliations:** 1 Department of Obstetrics and Gynecology, Guro Hospital, Korea University College of Medicine, Seoul 08308, Republic of Korea; mavrik8@naver.com; 2 Department of Obstetrics and Gynecology, Yonsei University College of Medicine, Seoul 03722, Republic of Korea; ponyata@naver.com (S.P.C.); ihlee86@yuhs.ac (I.L.); jhlee126@yuhs.ac (J.H.L.); yschoi08@yuhs.ac (Y.S.C.); 3 Department of Obstetrics and Gynecology, Gangnam Severance Hospital, Yonsei University College of Medicine, Seoul 06229, Republic of Korea; jggs95@naver.com (G.S.J.); sehee5515@gmail.com (S.K.); princessmin@hanmail.net (M.J.L.); imwooseok@yuhs.ac (W.I.); hmpark@yuhs.ac (H.P.); 4 Department of Medical Device Engineering and Management, Yonsei University College of Medicine, Seoul 06229, Republic of Korea; 5 Institute of Women’s Life Medical Science, Yonsei University College of Medicine, Seoul 03722, Republic of Korea; 6 Department of Obstetrics and Gynecology, Severance Hospital, Yonsei University College of Medicine, Seoul 03722, Republic of Korea

**Keywords:** formononetin, endometriosis, kip1 protein, p27, stat3 transcription factor, endometriosis mouse model

## Abstract

Objectives: Formononetin is one of the phytoestrogens that functions like a selective estrogen receptor modulator (SERM). In this study, we evaluated the effects of formononetin on endometriosis progression in vitro and in vivo. Materials and methods: After pathological confirmation, 10 eutopic and ectopic endometria were collected from patients with endometriosis. Ten eutopic endometria samples were collected from patients who did not have endometriosis. To determine the cytotoxic dose and therapeutic dose of formononetin, the concentration of 70% of the cells that survived after formononetin administration was estimated using a Cell counting kit-8 (CCK 8) assay. Western blot analysis was used to determine the relative expression levels of BAX, p53, pAKT, ERK, pERK, p27, and pSTAT3 in the eutopic endometria without endometriosis, eutopic endometria with endometriosis, and ectopic endometria with endometriosis as the formononetin concentration was increased. We confirmed the effect of formononetin on apoptosis and migration in endometriosis using fluorescence-activated cell sorting (FACS) and wound healing assays, respectively. A mouse model of endometriosis was prepared using a non-surgical method, as previously described. The mice were intraperitoneally administered formononetin for four weeks after dividing them into control, low-dose formononetin (40 mg/kg/day) treatment, and high-dose (80 mg/kg/day) formononetin treatment groups. All the mice were euthanized after formononetin treatment. Endometriotic lesions were retrieved and confirmed using hematoxylin and eosin (H&E) staining. Immunohistochemical (IHC) staining of p27 was performed. Results: We set the maximum concentration of formononetin administration to 80 μM through the CCK8 assay. Based on formononetin concentration, the expression levels of BAX, p53, pAKT, ERK, pERK, p27, and pSTAT3 proteins were measured using Western blot analysis (*N* = 4 per group). The expression level of pERK, p27, and pSTAT3 in eutopic endometrium with endometriosis tended to decrease with increasing formononetin concentration, and a significant decrease was noted at 80 μM. The expression of p27 in ectopic endometrium with endometriosis was also significantly decreased at 80 μM of formononetin. FACS analysis revealed that formononetin did not significantly affect apoptosis. In the wound healing assay, formononetin treatment revealed a more significant decrease in the proliferation of the eutopic endometrium in patients with endometriosis than in the eutopic endometrium without endometriosis. Relative expression of sex hormone receptors decreased with increasing formononetin doses. Although no significant differences were observed in the ER, PR-A, ERβ/ERα, and PR-B/PR-A, significant down-regulation of PR-B expression was noted after formononetin treatment at 80 μM. In the in vivo study, endometriotic lesions in the formononetin-treated group significantly decreased compared to those in the control group. The relative expression of p27 using IHC was highest in the control group and lowest in the high-dose formononetin treatment group. Conclusions: Formononetin treatment was shown to inhibit the proliferation of eutopic and ectopic endometria in patients with endometriosis through the regulation of p27, pSTAT3, and PR-B. In an endometriosis mouse model, formononetin treatment significantly reduced the number of endometriotic lesions with decreased p27 expression. The results of this study suggest that formononetin may be used as a non-hormonal treatment option for endometriosis.

## 1. Introduction

Endometriosis is a chronic gynecologic disease that pathologically defines the endometrial stromal and glandular tissue outside the uterus [1]. Endometriosis is found in approximately 10% of reproductive-aged women and is known to account for 25–40% of female infertility [2,3]. The etiology of endometriosis remains indefinite, but it is well known to be related to high estrogen levels and progesterone resistance [4,5].

Management of endometriosis requires consideration of subjective factors, such as patient symptoms, individual preferences and compliance, previous treatment, medical history, and family planning. Surgical treatment can destroy endometriotic lesions and dissect adhesive lesions, resulting in pain and subfertility. However, surgical treatment may not provide complete relief of symptoms, and it may induce a reduction in ovarian reserve [6].

Traditionally, hormonal treatment has been the primary treatment option for endometriosis. The main therapeutic strategy is to lower local or systemic estrogen levels. Progestins and oral contraceptives, as well as gonadotropin-releasing hormone (GnRH) agonists, are widely used to treat endometriosis-associated pain and prevent the recurrence of the disease after surgery [7]. Recently, GnRH antagonists have also been approved as a new treatment option for endometriosis [8]. Aromatase inhibitors, especially in post-menopause women [9], and COX inhibitors are another treatment option [10]. Approximately 50% of women diagnosed with endometriosis experience a recurrence of symptoms within 5 years despite proper treatment [11]. New treatments for endometriosis are being sought for various reasons, such as the recurrence of endometriosis despite appropriate treatment.

Phytoestrogens have received attention as potential therapeutic candidates for endometriosis. Phytoestrogens are compounds from plants that act as selective estrogen modulators in a tissue-specific manner [12]. Phytoestrogens can offer anti-proliferative, anti-inflammatory, antioxidant, and analgesic properties [13]. Specifically, formononetin, a phytoestrogen derived from *Pongamia pinnata*, *Astragalus membranaceus*, *Ononis angustissima*, and *Trifolium pratense* (*T. pratense*) [14], has been shown to have anti-tumor effects such as anti-proliferative, apoptosis-inducing, anti-cell migration, and anti-invasion effects on the breast, prostate, colon, nasopharyngeal, and lung cancer cells in vitro. Additionally, similar anti-tumor effects have been demonstrated under various tumor conditions in vivo experiments as well [15].

To date, no studies have reported the effects of formononetin on endometriosis pathogenesis. In this study, we evaluated the effects of formononetin on endometriosis and investigated its potential as a therapeutic agent for endometriosis using in vitro and in vivo experiments.

## 2. Materials and Methods

### 2.1. Study Population and Ethical Consideration

This study included 20 women. The patients underwent laparoscopic surgery for benign ovarian cysts at Gangnam Severance Hospital (Seoul, Republic of Korea) between 1 January 2018 and 31 December 2019. After pathological confirmation, patients were assigned to the study group. Ten patients were assigned to the endometriosis group. Eutopic and ectopic endometrial tissue samples were collected from patients with endometriosis. Ten patients without endometriosis diagnosed with benign ovarian cysts were assigned to the control group. Eutopic endometrial tissue samples without endometriosis were collected from patients of the control group.

This study was approved by the Institutional Review Board (IRB) of the Gangnam Severance Hospital, Yonsei University College of Medicine, Seoul, Republic of Korea (IRB approval number: 2021-0854-001). All patients in this study provided written informed consent, and the hospital’s ethics committees approved enrollment.

### 2.2. Formononetin

Formononetin has a molecular formula as follows: C16H12O4 (CAS no:485-72-3, IUPAC:7-hydroxy-3-(4-methoxyphenyl) chromen-4-one) and a molecular weight of 268.268 g/mol. It can be extracted from *Pongamia pinnata*, *Astragalus membranaceus*, *Ononis angustissima*, and *Trifolium pratense* (*T. pratense*) [14]. Formononetin was purchased from Sigma-Aldrich (St. Louis, MO, USA).

### 2.3. Primary Cell Culture

Each tissue sample was finely minced in Hank’s balanced salt solution containing hydroxyethyl piperazine ethane sulfonic acid (HEPES) (2 mmol/mL), penicillin (100 U/mL)/streptomycin (100 μg/mL), and collagenase (1 mg/mL, 15 U/mg). After 1 h of incubation at 37 °C, the dispersed cells were pelleted, washed, and resuspended in Dulbecco’s modified Eagle’s medium/Ham′s Nutrient Mixture F12 (DMEM/F-12) containing 10% fetal bovine serum (FBS) and 1% penicillin/streptomycin. After filtering using a 40 μm mesh cell strainer (Falcon, Corning, NY, USA), the cells were plated on 75 cm^2^ Falcon tissue culture flasks (BD Biosciences, Bedford, MA, USA). The analyses included cultured cells at 3–5 passages.

### 2.4. Cell Counting Kit-8 Assay (CCK 8 Assay)

After cell culture, eutopic endometrial cells from patients with and without endometriosis were assessed for cell viability and cytotoxicity using a cell counting kit 8 (CCK8) (Sigma-Aldrich). The cells in the exponential growth phase were seeded in 96-well plates at a density of 1 × 10^3^ cells/100 µL. After 24 h of culture at 37 °C, 0, 20, 40, 80, 100, and 200 µL of formononetin was added to each well. A total of 20 µL of CCK8 solution (CA1210-100, Beijing Solarbio Science & Technology, Co., Ltd., Beijing, China) was added to each well, including empty wells as controls. The optical density (OD) of each well was measured at 450 nm using a microplate reader (Bio-Rad 680; Bio-Rad Laboratories, Inc., Hercules, CA, USA) at 0.5, 1, 2, and 4 h after adding the CCK8 solution. The resulting OD was calculated by subtracting the control OD from the measured OD. The cell proliferation curves were plotted using these values.

### 2.5. Western Blot Analysis

Extraction of protein was carried out using radio-immunoprecipitation assay (RIPA) buffer (Thermo Fisher Scientific, Rockford, IL, USA) and protease and phosphatase inhibitor cocktail (Thermo Fisher Scientific, Rockford, IL, USA). Extracted protein concentration was measured using a bicinchoninic acid assay kit (Thermo Scientific, Waltham, MA, USA). A 20 μg protein sample was mixed with sodium dodecyl sulfate–polyacrylamide gel (SDS-PAGE) loading buffer (Biosesang, Seongnam, Gyeonggi, Republic of Korea). After heating at 95 °C for 5 min, protein sample was separated by SDS-PAGE. Then, protein sample was electrotransferred to a polyvinylidene fluoride membrane (Millipore, Burlington, MA, USA) using a transblot apparatus (Bio-Rad, Hercules, CA, USA). The polyvinylidene fluoride membranes (Millipore, Burlington, MA, USA) were blocked by 5% non-fat skim milk in Tris-buffered saline solution (10 mmol/L Tris-HCl (pH 7.4) and 0.5 mol/L NaCl) and 0.1% (*v*/*v*) Tween-20. The blots were probed with primary antibodies against the following proteins: phosphorylated protein kinase B (pAKT) (1:1000; Cell Signaling Technology, Danvers, MA, USA), BCL2 associated X (BAX) (1:1500, Cell Signaling Technology, Danvers, MA, USA) extracellular signal-regulated kinase (ERK) (1:1500 with 5% skim milk; Cell Signaling Technology, Danvers, MA, USA), phosphorylated ERK (pERK) (1:1000 with 5% skim milk; Cell Signaling Technology, Danvers, MA, USA), phosphorylation signal transducer and activator of transcription 3 (pSTAT3) (1:2000; Cell Signaling Technology, Danvers, MA, USA), Cyclin-dependent kinase inhibitor 1B (p27^Kip1^) (1:1000; Cell Signaling Technology, Danvers, MA, USA), p53 (1:1200, Cell Signaling Technology, Danvers, MA, USA), androgen receptor (AR) (1:400, Santa Cruz Biotechnology, Palo Alto, CA, USA), estrogen receptor (ERα) (1:100, Santa Cruz Biotechnology, Palo Alto, CA, USA), estrogen receptor (ERβ) (1:100, Santa Cruz Biotechnology, Palo Alto, CA, USA), progesterone receptor (PR-A) (1:200, Santa Cruz Biotechnology, Palo Alto, CA, USA), progesterone receptor (PR-B) (1:200, Santa Cruz Biotechnology, Palo Alto, CA, USA), and glyceraldehyde 3-phosphate dehydrogenase (GAPDH) (1:1000, Abcam, Cambridge, UK).

For secondary antibody, a horseradish peroxidase-conjugated anti-mouse or anti-rabbit antibody (1:2000; Thermo Fisher Scientific) was used. Enhanced chemiluminescence (Santa Cruz Biotechnology, Dallas, TX, USA) was used for protein detection. All experiments were performed in triplicates. Relative OD of blot was measured by ImageQuant LAS 4000 (GE Healthcare, Little Chalfont, UK), and the blot images were analyzed using ImageJ software (https://imagej.nih.gov/ij/, version 1.53k; Java 1.8.0_172, Wayne Rasband, US National Institutes of Health) accessed on 1 March 2022.

### 2.6. Fluorescence-Activated Cell Sorting (FACS)

FACS (BD Cell Quest^®^ version 3.3) was carried out using an Annexin V-Fluorescein isothiocyanate (FITC) Early Apoptosis Detection Kit (Cell Signaling Technology, Inc.). This kit included Annexin V-FITC and propidium iodide (PI) to confirm changes in the apoptotic populations of eutopic endometrial cells from patients with and without endometriosis treated with formononetin. Eutopic endometrial cells from patients with and without endometriosis were cultured and treated with 0, 80 μM formononetin for further 24 h. The plate on which the cells adhered was rinsed with Dulbecco’s phosphate-buffered saline (DPBS), and cells were detached using trypsinization. The cells underwent resuspension in 1X binding buffer (BD Biosciences). An amount of 1 µL Annexin V-FITC and 12.5 µL PI were added to 96 µL cell suspension. After incubation for 15 min at 37 °C, each apoptotic cell population was analyzed by the BD Cell Quest^®^ version 3.3. software (Becton Dickinson and Company, Franklin Lakes, NJ, USA).

### 2.7. Wound Healing Assay

Eutopic endometrial cells from patients with and without endometriosis were seeded in 6-well culture plates (5.0 × 10^5^ cells/well) containing DMEM/F12, 10% FBS, and antibiotics with 0 or 80 μM formononetin. The culture plates were placed in a humidified atmosphere containing 5% CO_2_/95% air at 37 °C for 24 h. A linear wound was made by a sterile 200 µL pipette tip. After washing the debris with phosphate-buffered saline (PBS) and adding culture media, the culture plates were incubated for 24 h at 37 °C with 5% CO_2_. The images obtained by EVOS inverted microscope (Advanced Microscopy Group, Mill Creek, WA, USA) were analyzed using ImageJ software with the following calculation: [(mean wound width × mean remaining width)/mean wound width × 100 lrb%].

### 2.8. Mouse Model of Endometriosis and Ethical Consideration

All procedures were carried out in accordance with the National Institutes of Health Guide for the Care and Use of Laboratory Animals. This in vivo study was approved by the Institutional Animal Care and Use Committee of Gangnam Severance Hospital, Yonsei University College of Medicine, Seoul, Republic of Korea (Protocol number: 2021-0220).

Healthy female C57BL/6 mice (seven weeks of age) were imported from Orient Bio Inc. (Seongnam, Gyeonggi, Republic of Korea). The mice were randomly assigned. They were housed in standard clear plastic cages, with five mice in each cage under a 12:12 h light/dark cycle at 21 °C with ad libitum access to water and food. Euthanasia was induced by CO_2_ inhalation, and an effort was made to minimize the suffering of the animals in all procedures. A mouse model of endometriosis was established using a non-surgical method. Uterine tissues were obtained from 15 donor mice that had grown endometria by subcutaneous injection of 3 mg estradiol benzoate, sliced to <1 mm in diameter, and injected intraperitoneally into 30 recipient mice, as previously described [16].

One week after endometriosis induction, the mice were randomly assigned to three groups: control (0 mg/kg/day) group (*N* = 10), low-dose formononetin (40 mg/kg/day) group (*N* = 10), and high-dose formononetin (80 mg/kg/day) group (*N* = 10). The dose of formononetin administered in this study was set with reference to the dose administered in previously published in vivo tumor-bearing animal models [15]. Formononetin was injected intraperitoneally daily. After four weeks of formononetin treatment, no difference was observed in the general appearance of all three groups. All 30 mice were euthanized for the confirmation and retrieval of endometriotic lesions. Endometriotic lesions included implants and cysts. These values were based on the length and width of each lesion. The implants were retrieved, fixed in 10% formalin-acetic acid, and embedded in paraffin. Hematoxylin and eosin staining of paraffin-embedded sections was carried out to confirm endometriosis. Immunohistochemical staining was performed to confirm p27 expression.

### 2.9. Immunohistochemistry (IHC)

After deparaffinization with xylene and dehydration with an alcohol gradient, 3% H_2_O_2_ was applied for 10 min to inactivate endogenous peroxidase activity. After incubation with 10% normal goat serum in PBS for 1 h at room temperature to block non-specific binding, the slides were incubated with primary antibody p27 overnight at 4 °C, followed by biotinylated goat anti-rabbit IgG (Sigma, MO, USA) for 1 h at room temperature. A streptavidin–biotin–peroxidase complex assay was carried out. The expression of p27 was analyzed by ImageJ software with the IHC Image Analysis Toolbox. Briefly, IHC toolbox identified the chromogen-stained areas and assigned them to the region of interest (ROI). The IHC-stained ROI was darker and had values of <255, and unstained or white areas in the RGB images had a maximum value of 255.

IHC staining intensity = [(255 − ROI stain score) × %ROI (ROI pixels/total image pixels × 100)].

### 2.10. Statistical Analysis

Data are presented as means ± standard deviation (SD). Data from in vitro experiments were analyzed using Kolmogorov–Smirnov or Shapiro–Wilk tests to investigate whether they were normally distributed. Student’s *t*-test or the Mann–Whitney U test was used to evaluate the difference between groups. The in vivo data were analyzed using the one-way analysis of variance to investigate differences between three groups, followed by Bonferroni’s post hoc analysis. All statistical analyses were performed using SPSS 25.0 software (SPSS Inc., Chicago, IL, USA). A *p*-value < 0.05 was defined as statistically significant.

## 3. Results

### 3.1. Formononetin Affected Endometrial Cell Viability

**Figure 1:**
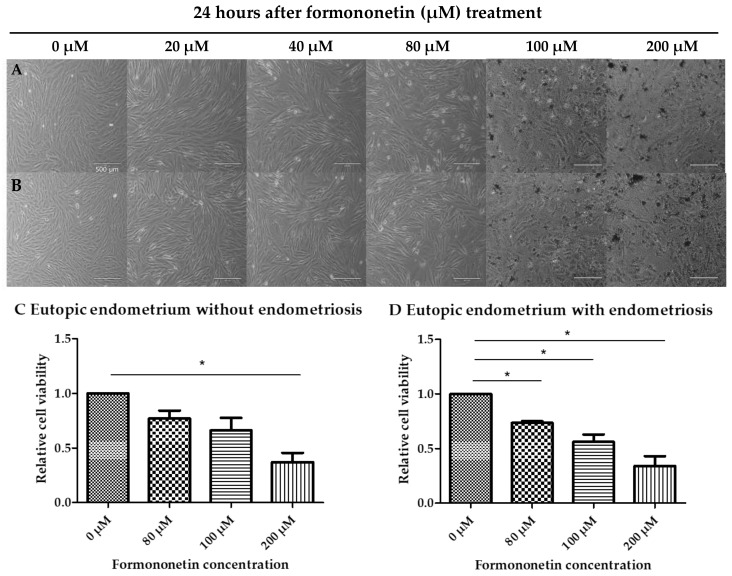
CCK8 assay showed decreased cell viability in eutopic endometrium without endometriosis (**A**,**C**) and eutopic endometrium with endometriosis (**B**,**D**) by increasing dose of formononetin (×40 magnification, bar scale 500 μm, * *p* < 0.05, *N* = 3 per group).

The CCK8 assay was carried out to investigate the cytotoxicity of formononetin in eutopic endometrial cells from patients with and without endometriosis. Formononetin decreased the cell viability in a dose-dependent manner in both endometrial cell lines. The decrease in cell viability was statistically significant over 80 μM in eutopic endometrial cells from patients with endometriosis (1 vs. 0.73 ± 0.02, *p* = 0.013) and over 200 μM in eutopic endometrial cells without endometriosis (1 vs. 0.37 ± 0.15, *p* = 0.02) (Figure 1). In this study, we set the maximum dose at 80 μM with a cell viability of 70% when formononetin was used.

### 3.2. Formononetin Decreased Expression of p27 and pSTAT3 on Eutopic Endometrium with Endometriosis and p27 of Ectopic Endometrium with Endometriosis in Dose-Dependent Manner

**Figure 2:**
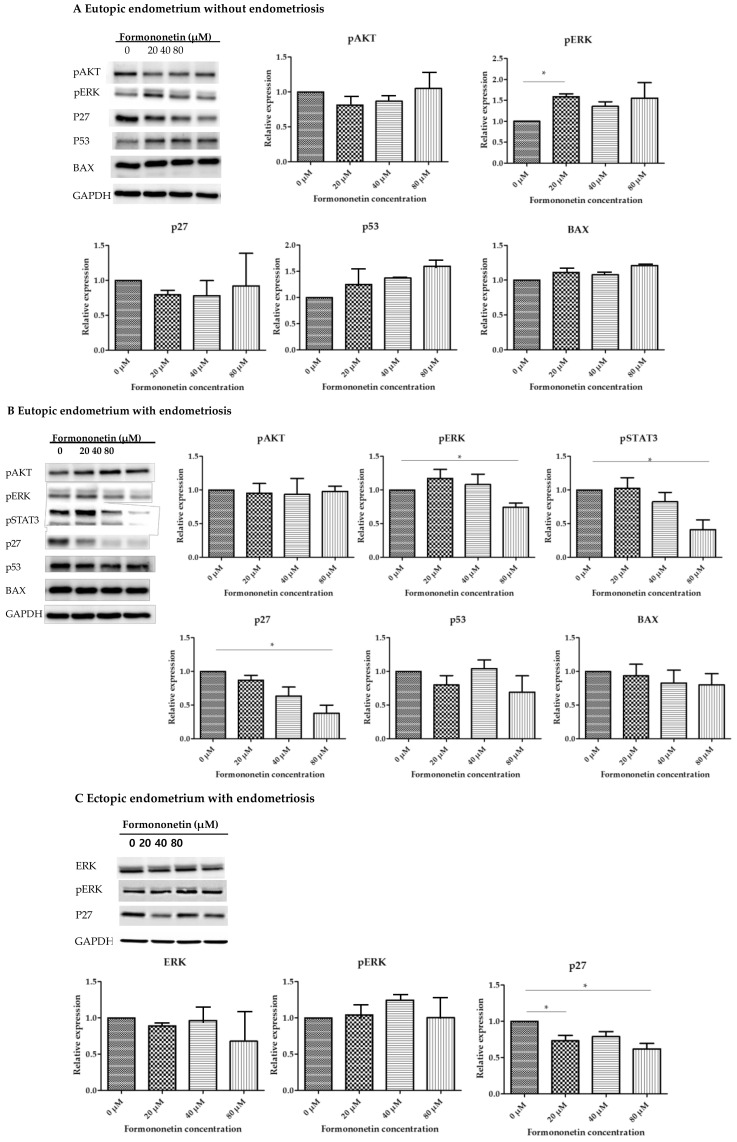
Band of Western blot of BAX, p53, pAKT, pERK, and p27 in eutopic endometrium without endometriosis (**A**), BAX, p53, pAKT, pERK, p27, and pSTAT3 in eutopic endometrium with endometriosis (**B**), and ERK, pERK, and p27 in ectopic endometrium with endometriosis (**C**). Relative expression level of BAX, p53, pAKT, ERK, pERK, p27, and pSTAT3 was described in a box plot. Relative expression levels of BAX, p53, pAKT, and ERK with increased formononetin concentration did not show a statistically significant trend in 3 groups. Relative expression level of p27 decreased statistically significantly in dose-dependent manner in eutopic endometrium and ectopic endometrium with endometriosis, not in eutopic endometrium without endometriosis (* *p* < 0.05, *N* = 4 per group).

Figure 2 shows the relative expression levels of BAX, p53, pAKT, ERK, pERK, p27, and pSTAT3 in eutopic endometria with and without endometriosis, and in ectopic endometria with endometriosis, after formononetin treatment.

In eutopic endometria without endometriosis, no significant differences were observed in the expression of BAX, p53, pAKT, or p27 after formononetin treatment. Only pERK showed higher expression levels in the 20 μM treatment group than in the control group with statistical significance (1 vs. 1.6 ± 0.11, *p* = 0.01).

In the eutopic endometrium of patients with endometriosis, no significant differences were observed in the expression of BAX, p53, or pAKT after formononetin exposure. However, the expressions of pERK, pSTAT3, and p27 showed a tendency to decrease with increasing formononetin concentration, and statistical significance had reached 80 μM compared with the control group (1 vs. 0.74 ± 0.12, *p* = 0.02, 1 vs. 0.41 ± 0.29, *p* = 0.03, and 0.38 ± 0.24, *p* = 0.01, respectively).

The relative expression of ERK and pERK in ectopic endometria with endometriosis did not show any increasing or decreasing trend with increasing formononetin treatment dose. However, the relative expression of p27 significantly decreased in the 20 μM and 80 μM treatment groups than in the no treatment group (1 vs. 0.73 ± 0.15, *p* = 0.04, and 1 vs. 0.62 ± 0.15, *p* = 0.01, respectively).

### 3.3. Formononetin Did Not Affect Apoptosis in Eutopic Endometrium with and without Endometriosis

**Figure 3:**
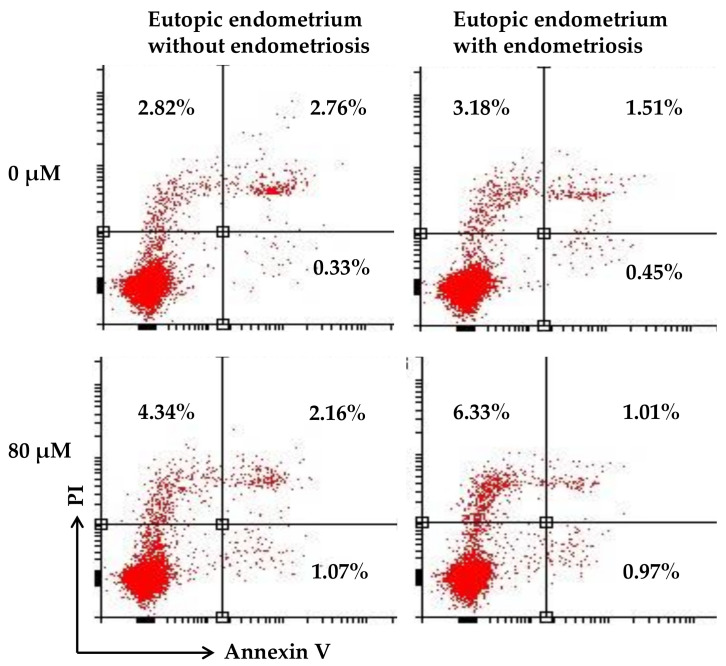

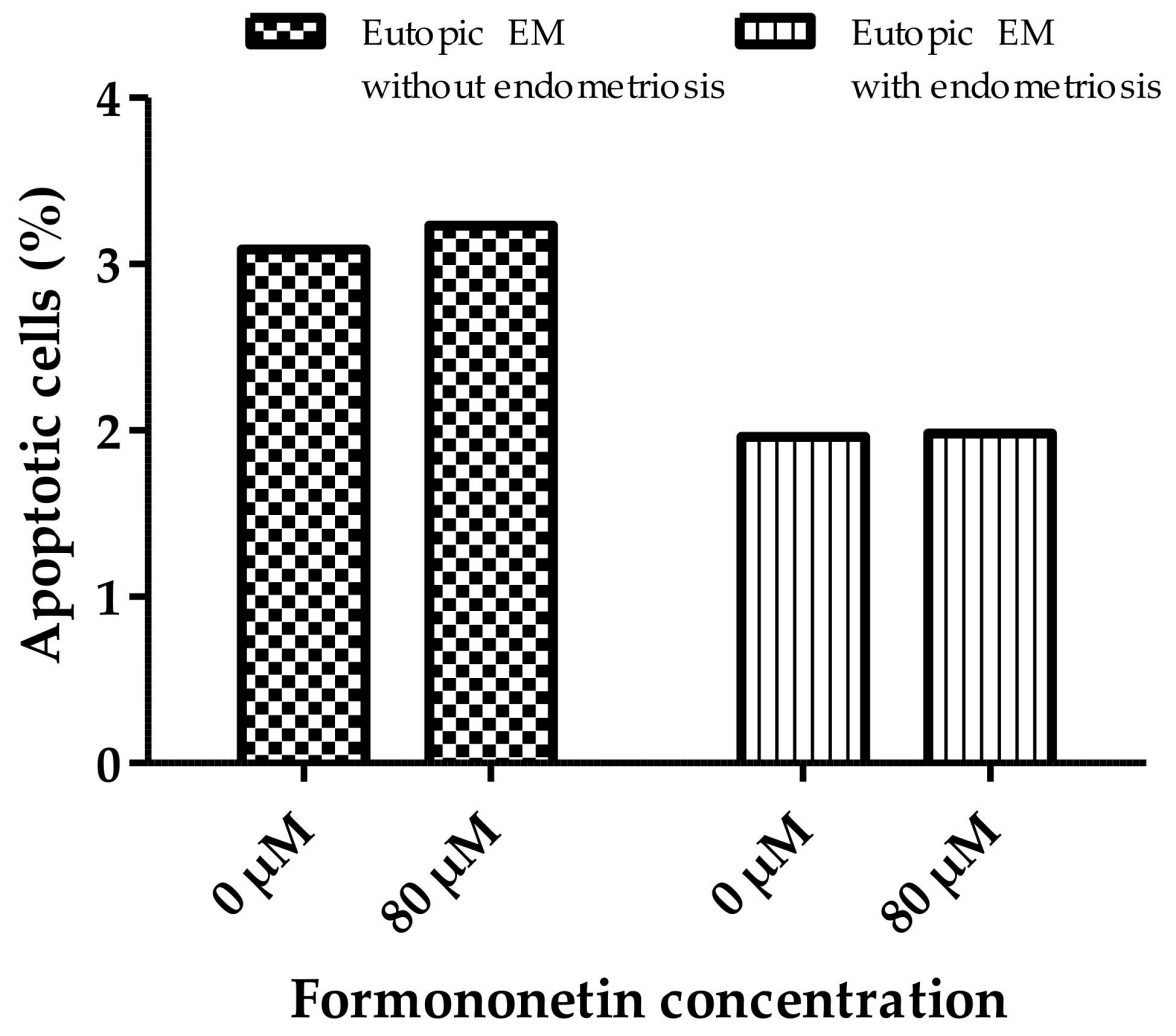
Formononetin did not affect apoptosis on eutopic endometrial cells with and without endometriosis.

Annexin V- and PI-based apoptosis and necrosis discrimination assays were performed on eutopic endometria with and without endometriosis. The results showed that formononetin did not significantly induce apoptosis in eutopic endometrial cells with or without endometriosis in a dose-dependent manner (Figure 3).

### 3.4. Formononetin Inhibits Cell Migration in Eutopic Endometrium with and without Endometriosis

**Figure 4:**
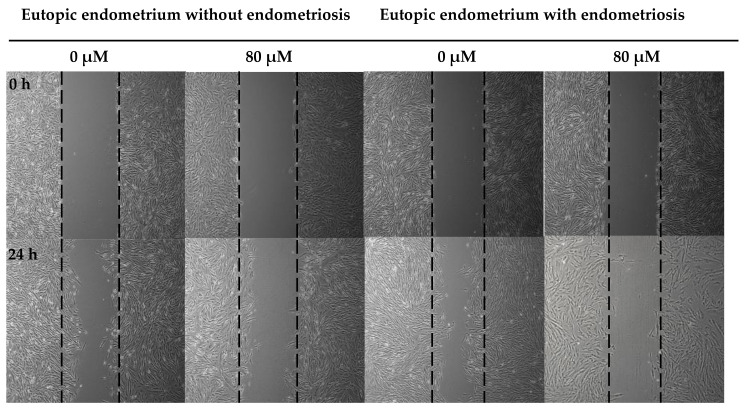

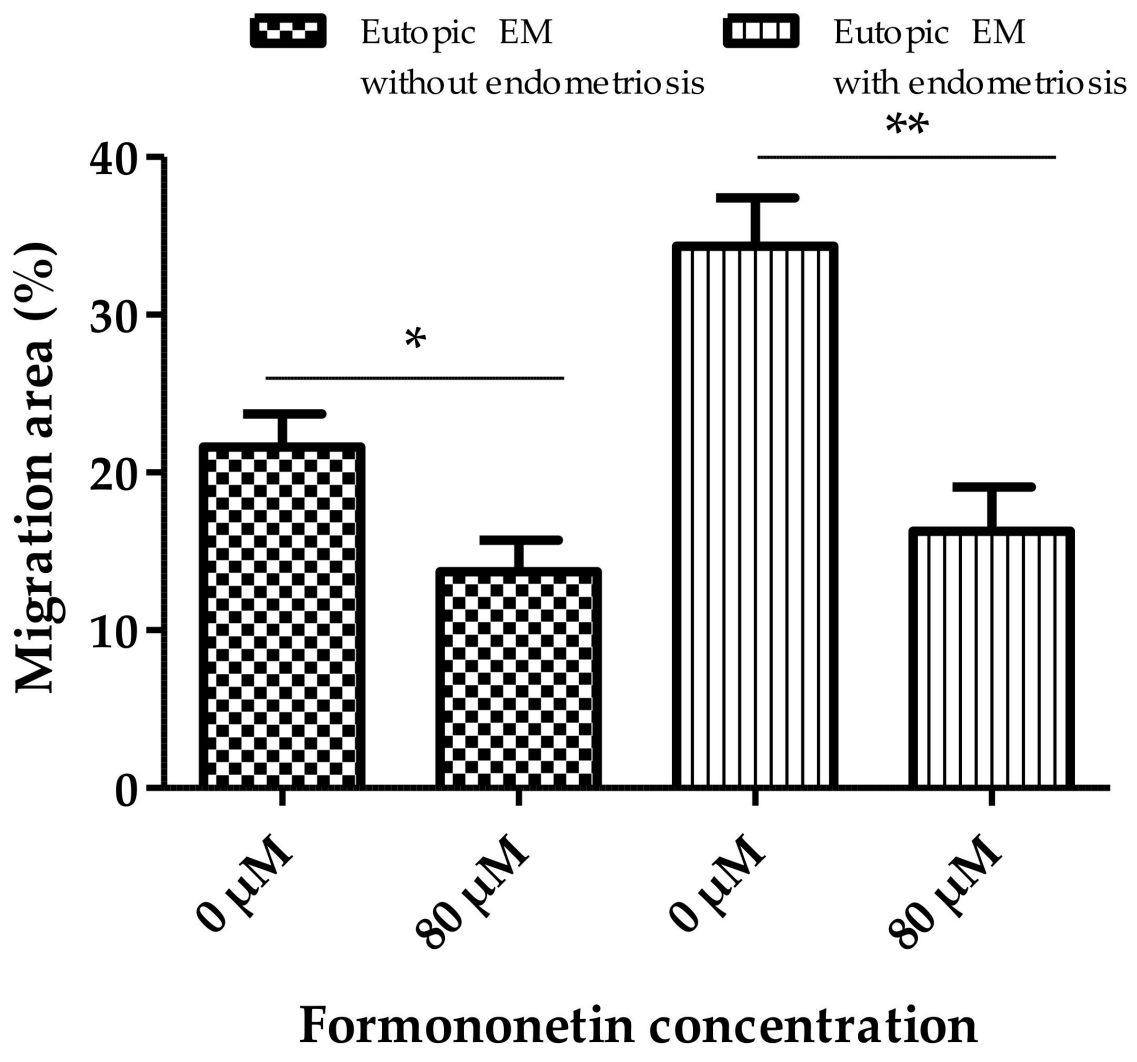
Formononetin affects cell migration in eutopic endometrial cells with and without endometriosis, but has more anti-proliferative effect in eutopic endometrium with endometriosis in wound healing assay (×40 magnification, * *p* < 0.05, ** *p* < 0.01, *N* = 3 per group).

A wound healing assay was performed to investigate the anti-migratory effect of formononetin. In eutopic endometria without endometriosis, administration of formononetin induced a significant decrease in wound closure (21.62 ± 6.27 vs. 13.72 ± 5.99, *p* = 0.047). In eutopic endometrium with endometriosis, the reduction in wound closure was more prominent than control endometrium with statistical significance (34.37 ± 8.61 vs. 16.28 ± 8.45, *p* = 0.001) (Figure 4).

### 3.5. Formononetin Decreased Expression of Progesterone Receptor-B (PR-B) on Eutopic Endometrium with Endometriosis in Dose-Dependent Manner

**Figure 5:**
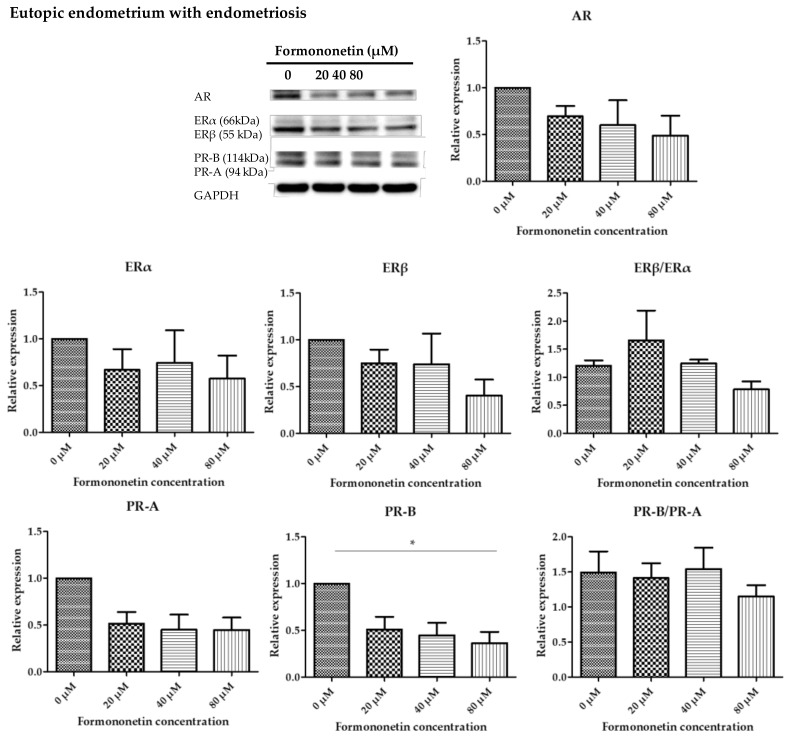
Relative expression levels of androgen receptor (AR), estrogen receptor (ER), progesterone receptor (PR), ERβ/α, and PR-B/A in eutopic endometrium with endometriosis had decreasing tendency, but there is statistical significance only in PR-B (* *p* < 0.05, *N* = 3 per group).

Figure 5 shows the difference in the relative expression level of sex hormone receptors, including androgen receptor (AR), estrogen receptor alpha (ERα), estrogen receptor beta (ERβ), progesterone receptor-A (PR-A), progesterone receptor-B (PR-B), ERβ/ERα, and PR-B/PR-A with increased formononetin concentration in eutopic endometrium with endometriosis. Although the relative expression of each hormone receptor tended to decrease with increasing formononetin, no significant difference was observed in AR, ERα, ERβ, PR-A, ERβ/ERα, and PR-B/PR-A. In contrast, significantly decreased expressions of PR-B were noted after formononetin treatment at 80 μM (0.36 ± 0.21, *p* = 0.03).

### 3.6. Mouse Model of Endometriosis

**Figure 6:**
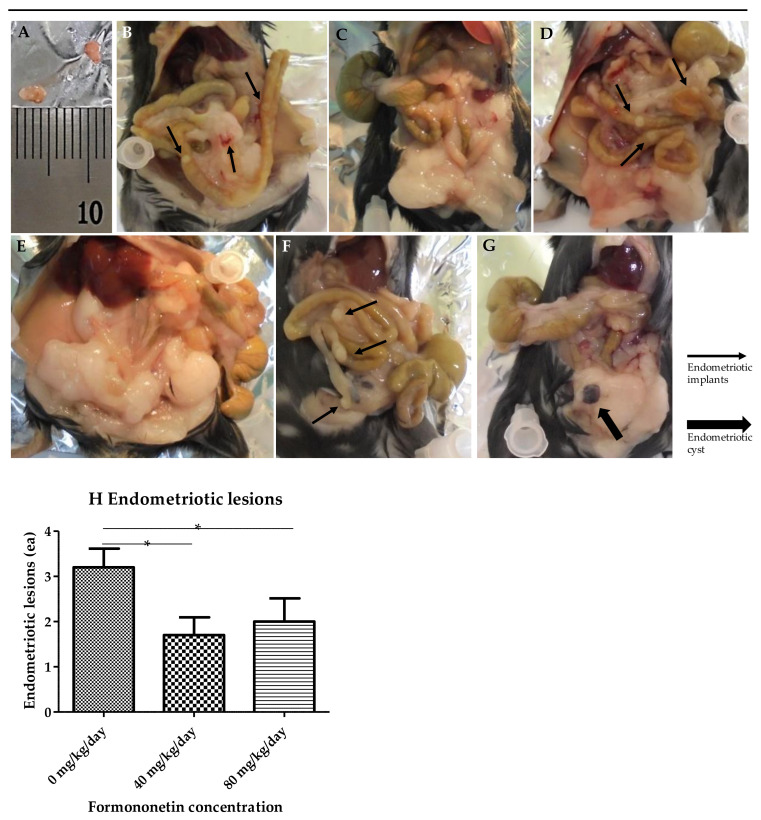
Mouse endometriosis model results (**A**–**H**). Photographs showing endometriotic implants in mouse model in control, low dose (40 mg/kg/day), and high dose (80 mg/kg/day) of formononetin for 4 weeks. (**A**) The size of endometriotic implants of 3 groups was similar (4 mm × 2 mm). (**B**) Control group with endometriotic implants (narrow arrow). (**C**) Low-dose (40 mg/kg/day) group without endometriotic implants. (**D**) Low-dose group with endometriotic implants (narrow arrow). (**E**) High-dose (80 mg/kg/day) group without endometriotic implants. (**F**) High-dose group with endometriotic implants (narrow arrow). (**G**) High-dose group with endometriotic cysts (4 mm × 4 mm) (wide arrow). (**H**) Endometriotic lesions (implants and cysts) (* *p* < 0.05, *N* = 10 per group).

**Figure 7:**
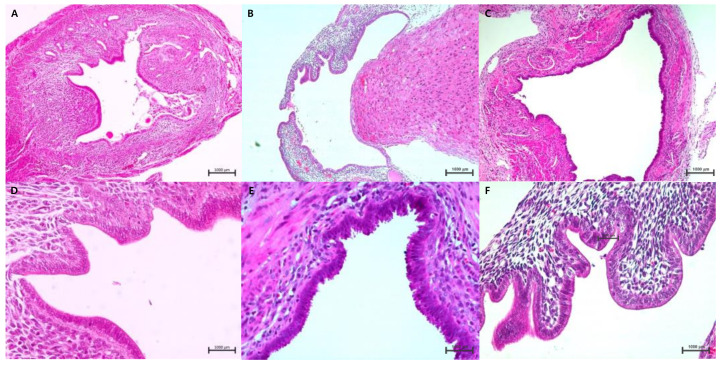
H&E staining of the endometriotic implants of each treatment group ((**A**–**C**): ×10 magnification, (**D**–**F**): ×400 magnification). Dilated glands lined a single layer of columnar cells with surrounding stroma were confirmed in all three groups. (**A**,**D**) control group, (**B**,**E**) low-dose formononetin treatment group (40 mg/kg/day), (**C**,**F**) high-dose formononetin treatment group (80 mg/kg/day).

After formononetin treatment, 30 recipient mice were euthanized to confirm and collect endometriotic lesions. The endometriotic lesions were evaluated based on the number of implants (ea) rather than the area (ex. mm^2^) because all three groups had similar endometriotic implants (4 mm width × 2 mm length). An average of 3.2 ± 0.4 (1–5) endometriosis implants (ea) occurred in the control group not treated with formononetin. In the low-dose group, an average of 1.7 ± 0.4 (0–4) endometriotic implants were found in the low-dose group, which was significantly lower than that in the control group. In the high-dose group, an average of 2.0 ± 0.5 (0–6) endometriotic implants were identified in the high-dose group, which was significantly lower than that in the control group; however, it did not differ significantly from the low-dose group. The high-dose group showed greater heterogeneity than the other groups, ranging from zero to six implants. Furthermore, the formation of a characteristic chocolate-colored fluid containing an endometriotic cyst measuring 4 mm × 4 mm was also confirmed in one case (Figure 6). Retrieved endometriotic implants from the three groups were stained with H&E to confirm the presence of endometrial glands and stroma. The endometrial glands and stroma were observed in all three groups (Figure 7).

**Figure 8:**
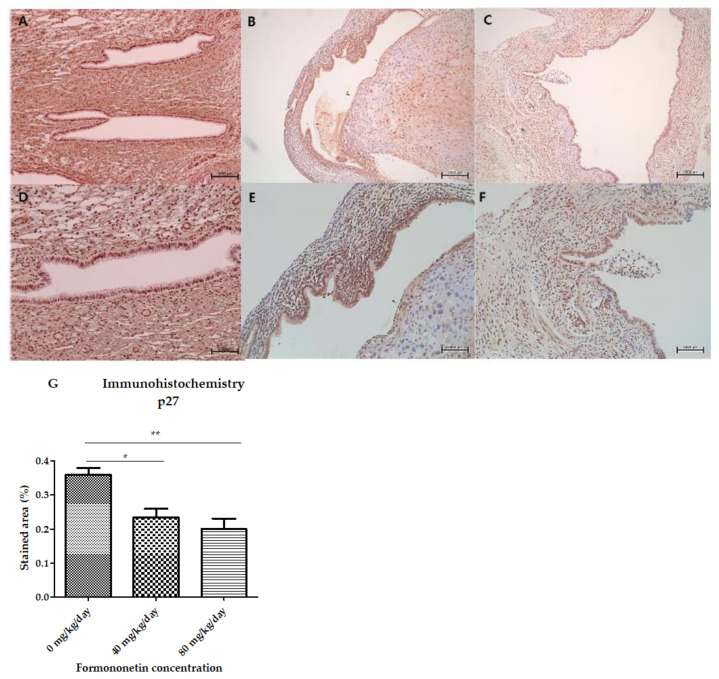
Immunohistochemistry staining for p27 expression of the endometriotic implants of each treatment group: (**A**,**D**) control group, (**B**,**E**) low-dose group (40 mg/kg/day), (**C**,**F**) high-dose group (80 mg/kg/day). ((**A**–**C**): ×100 magnification, (**D**–**F**): ×200 magnification). (**G**) The relative expression of p27 was highest in the control group and lowest in the high-dose formononetin treatment group (* *p* < 0.05, ** *p* < 0.01).

We performed IHC staining to investigate the expression of p27, which showed a statistically significant trend with increasing formononetin concentrations in vitro. The relative expression of p27 was the highest in the control group and lowest in the high-dose formononetin treatment group (0.36 ± 0.04 vs. 0.23 ± 0.05 vs. 0.20 ± 0.05, control vs. low dose; *p* = 0.03, control vs. high dose; *p* = 0.002) (Figure 8).

## 4. Discussion

This study aimed to evaluate the effects of formononetin on endometriosis and explore its potential as a therapeutic agent for endometriosis using in vitro and in vivo experiments. In this study, we demonstrated that formononetin treatment effectively suppressed the proliferation of endometrial cells in endometriosis at appropriate concentrations, which appeared to be mediated through the downregulation of p27, pSTAT3, and PR, especially PR-B expression.

In this study, we set the maximum concentration of formononetin to 80 μM in vitro using the CCK8 assay. Western blot was performed to identify the expression of pAKT, p27, and pSTAT3 in relation to proliferation and BAX, ERK, pERK, and p53 in relation to apoptosis using previous cancer studies as reference [17]. When treated with formononetin, the expression of p27 in the eutopic and ectopic endometria of patients with endometriosis significantly decreased in a dose-dependent manner. The expression of pERK showed a significant decrease in eutopic endometrium with endometriosis at 80 μM formononetin treatment but did not show a specific trend in ectopic endometrium with endometriosis. On the other hand, the expression of p27 showed a significant reduction in both eutopic and ectopic endometria with endometriosis at 80 μM formononetin treatment. This means that ectopic endometrium with endometriosis may act in some different molecular pathways than eutopic lesions in the process of engraftment outside the uterus. Interestingly, the expression levels of hormone receptors also changed significantly following formononetin treatment. The expressions of ER, PR, and AR, as well as ERβ/ERα and PR-B/PR-A ratios, all decreased with increasing formononetin concentration, with a statistically significant decrease in PR-B expression. Our findings in the wound healing assay indicate that formononetin may have an anti-migratory effect on the eutopic endometrium in patients with endometriosis, although the flow cytometry results show that its effect on apoptosis is minor. In the in vivo study, formononetin reduced endometriotic lesions compared to those in the non-treated control group. When the relative expression levels of p27 were evaluated, the highest formononetin dose treatment group had the lowest expression, followed by the low-dose formononetin treatment group and the non-treated group.

The *CDKN1B*-encoded protein p27, also known as KIP1, binds to cyclin-dependent kinases (Cdks) and regulates G0 to S phase transition [18]. It is not a classic tumor suppressor like p53, but has been examined in various tumor types [19]. This protein may positively or negatively influence cell proliferation, cell motility, and apoptosis [20]. Deregulated cytoplasmic p27 could activate cell motility and invasion, and cause tumor progression [21]. p27 is also known as a key regulator of endometriosis [22]. However, a previous study reported significantly decreased levels of p27 in endometrial cells in patients with endometriosis compared to those without the disease. The expression of p27 in the endometriosis group increases according to the severity of the disease [23]. Additionally, 57.7% of patients with endometriosis showed p27 expression, compared to 50.0% of patients without endometriosis [24]. Moreover, increased expression of p27 contributes to the progression of ectopic lesions such as peritoneal endometriosis [25]. Therefore, we believe that the effect of formononetin on the reduction in endometriotic lesions in a mouse model was achieved via the reduction in increased expression of p27 in endometriosis.

Our in vitro results indicated that pSTAT3 expression was significantly reduced by formononetin treatment. STAT3, a member of the STAT (signal transducer and activator of transcription pathway) family, was phosphorylated by Janus kinase (JAK), and it could promote proliferation, angiogenesis, and regulate tumor microenvironment [26,27]. Regardless of the proliferative or secretory phase, eutopic endometrium with endometriosis was found to increase pSTAT3 expression more than eutopic endometrium without endometriosis [28]. Tofacitinib, a JAK inhibitor, showed potential as a therapeutic agent for endometriosis by reducing pSTAT3 expression and the volume of endometriosis lesions in the endometriosis mouse model [29]. Interestingly, formononetin has potential effects in endometriosis via the alteration of pSTAT3 expressions. Notably, our results indicate a possible link between p27 and STAT3 in endometriosis. To the best of our knowledge, no study has evaluated the relationship between p27 and pSTAT3 expression in endometriosis. Previous studies have indicated the possible roles of p27 and STAT3 in malignant conditions. In a study of immortalized human mammary epithelial MCF-12A cell lines, p27 upregulated STAT3, promoting cell migration [30]. Endometriosis is not a malignant condition, but it can form ectopic lesions like cancer. Therefore, cell migration is thought to be important in the progression of endometriosis [2]. Similarly, our results showed that formononetin treatment reduced p27 expression in the eutopic and ectopic endometrium and pSTAT3 expression in the eutopic endometrium of patients with endometriosis, thereby explaining the potential therapeutic effects of formononetin in endometriosis.

Our study also revealed an intriguing effect of formononetin on sex steroid hormone receptors. Phytoestrogen may serve as a selective progesterone receptor modulator (SPRM), operating as an agonist or antagonist depending on the target tissue, though its precise impact and mechanism on certain tissues are still poorly known [31]. To our knowledge, this is the first study to evaluate the effects of formononetin on sex hormone receptors in the human endometrium. Our findings suggest that formononetin functions as an anti-progestin agent in the eutopic endometrium of patients with endometriosis. Formononetin effectively reduced PR-B expression in the eutopic endometria of patients with endometriosis. These results contrast previous research on dienogest (17a-cyanomethyl-17b-hydroxyestra-4,9-dien3-one), which is frequently used to treat endometriosis and alleviate progesterone resistance by elevating PR-B, PR-B/PR-A ratio and lowering ERβ/ERα ratio [32]. We believe that formononetin has a different mechanism of action on progesterone receptors in endometriosis.

These results may be secondary to a decrease in estrogen receptor expression. A relatively high level of ERβ in endometriosis is known to inhibit the induction of estradiol-dependent PR by suppressing Erα [33]. Notably, PR-B undergoes post-translational modification and has a rapid turnover, making PR-B difficult to detect [34]. The ERβ level of endometriosis stromal cells was 142-fold higher than normal stromal cells, while the ERα level of endometriosis stromal cells was 9-fold lower than normal stromal cells. An abnormally high ERβ/ERα ratio reduces PR expression by derangement by estradiol induction. Progesterone resistance indicates low PR expression [35,36].

Phytoestrogens are known to act through estrogen receptors because of their similar chemical structures. They have a lower affinity than estrogen for binding to both ERα and ERβ. Estradiols show similar affinity to ERα and ERβ, but phytoestrogens show higher affinity to ERβ than to Erα [37]. Several studies were conducted to investigate the effect of phytoestrogen on endometriosis treatment, including a total of seven human studies. One study found no change in endometriosis pain, one reported a reduced risk of endometriosis, and five studies reported endometriosis pain relief [38].

Female mice in the reproductive period are known to have periodic fluctuations in hormone levels [39]. The action of hormone receptors varies between species, and it is known that hormone receptor mutations are more critical in humans than in rodents [40]. Therefore, the experiment for the hormone lab and their receptor expression was not carried out in the in vivo study. If the hormone measurements had been taken multiple times per individual mouse, we might find that formononetin treatment affects hormonal levels.

There were fewer endometriotic implants in the formononetin 40 mg/kg group than in the 80 mg/kg group, but the difference between the two groups was not statistically significant. This study was initiated in the absence of a reference value for formononetin in an endometriosis animal model. In the future study, it will be necessary to confirm by fine-tuning whether the corresponding concentration range is the threshold that has the therapeutic effect for endometriosis.

In conclusion, this study is the first to evaluate the effects of formononetin on endometriosis. Formononetin revealed an anti-proliferative effect in endometriosis in the in vitro study. It may have been due to the regulation of p27, pSTAT3, and PR-B. In a mouse model of endometriosis, formononetin reduced the number of endometriotic lesions with decreased p27 expression. Our results suggest that formononetin may have therapeutic potential in endometriosis. Further studies are warranted to elucidate the underlying therapeutic mechanisms of formononetin and its effects in clinical settings.

## Data Availability

The data presented in this study are available on request from the corresponding author.

## Abbreviations

AR: Androgen receptor; BAX, BCL2 associated X; CCK8, Cell counting kit 8; ERK, extracellular signal-regulated kinase, ER, Estrogen receptor; GAPDH, glyceraldehyde 3-phosphate dehydrogenase; pAKT, phosphorylated protein kinase B; pERK, phosphorylated ERK; PR, Progesterone receptor; pSTAT3, phosphorylation-signal transducer and activator of transcription 3, p27^Kip1^, Cyclin-dependent kinase inhibitor 1B.
